# The Re-Introduction of Small Pox.—II

**Published:** 1898-09-10

**Authors:** 


### Modern Sociology.

### THE RE-INTRODUCTION OF SMALL-POX.—II.

We showed in a former paper that, on the occurrence of an outbreak of small-pox in a locality unprotected by vaccination, or containing a considerable number of persons whose protection had been imperfect and inadequate, there would be every reason to anticipate an almost universal prevalence and a terrible mortality. No history handed down from the Middle Ages displays a greater malignity of the disease than that which was experienced in France during and after the war with Germany; and we have seen that the mortality in Gloucester amounted, in round numbers, to almost a fourth of those who were attacked. We have to-day to regard the question from the point of view of the frequently crippled and scarred survivors, who would too often be ready to think with some envy of the fate of those who had been released by death from a future of disfigurement and misery. Macaulay, in his account of the long courtship between Sir William Temple and Dorothy Osborne, tells us that, when the lovers had triumphed over all the obstacles which kinsmen and rivals could oppose, a yet more serious calamity befell them. "Poor Mistress Osborne fell ill of the small-pox, and, though she escaped with life, lost all her beauty." To this most severe trial, he continues, "the affection and honour of the lovers of that age were not unfrequently subjected." Quite lately a distinguished artist has quoted in the *Times* the testimony of an old gentleman whom he knew in his youth, and whose opinion he asked as to the comparative beauty of women in this and the preceding century. The reply was that women in the last century were so constantly disfigured by small-pox that anyone who escaped, and who therefore retained her natural complexion and features, was then regarded as a beauty. The inexpressible loathsomeness of the disease during its acute stage, and the sickening odour by which it is attended, have quite faded from the knowledge of the present generation. But, as regards survivors, unquestionably the most important consequence is the production of blindness. After an attack of small-pox, a girl never recovers her complexion; but it often happens that time does a great deal to efface the scars, and, in a population among which nearly all the girls had suffered, there can be no doubt that the attractions of sex, in the great majority of cases, would triumph over the loss of beauty. But loss of sight, when occasioned by small-pox, is irremediable, depending, as it usually does, upon ulceration of the cornea, and the consequent formation of a dense and opaque cicatrix. Dr. Brailey has published an account of sixteen persons who applied as patients at the Royal London Ophthalmic Hospital, in consequence of having lost one or both eyes from small-pox. Of these persons not a single one had been revaccinated, nor, indeed, had a single one been vaccinated to the extent required of vaccinators under contract with the Local Government Board. Judged by these figures, collected ten years ago, the neglect or the perfunctory performance of vaccination might even then be credited with 2·1 per cent. of the cases in England in which an eye had been lost. Two years previously, Magnus investigated the causes of blindness, of both eyes, in 3,204 persons of both sexes, under the age of 20, who were being taught at one or other of 64 European institutions for the instruction of the blind. Among them, 240 persons, or 7·49 per cent. of the whole, had been blinded by small-pox. In this country, now for many years, small-pox has been very slightly operative as a cause of blindness; and it cannot be questioned that those who are responsible for the re-introduction of such a scourge must indeed have hearts like the nether millstone if they are able to contemplate the probable results of their handiwork without some disturbance of mind.

We return to the question of what can be done by rational people to protect themselves against the folly of the "conscientious objector," the abominable wickedness of the merely venal agitator, the deplorable weakness of the Government, and the still more deplorable subserviency of Parliament. It must be remembered that the "conscience" question, as a rule, is only skin-deep. As we formerly mentioned, during the course of the Gloucester epidemic no less than 36,000 persons, the majority of them "conscientious" anti-vaccinators, rushed off to the various stations to be vaccinated; and we lately heard a droll instance of a gentleman who paraded his objections with great vigour at an insurance office, until he found that his unvaccinated condition involved a considerable addition to the annual premium for his life policy. He then explained that, after all, the objection was rather on his father's part than on his own; and he ultimately sent in a satisfactory certificate of having successfully undergone the operation.

The problem which now confronts English people is not quite a new one, something of the same kind having been dealt with by our forefathers in relation to the now forgotten, but once very real, prevalence of leprosy. In old times, the subjects of a loathsome and infectious disease were confined within certain limits, and were not permitted unrestricted intercourse with their fellow-citizens. Even in public worship they were held apart; and there are churches in which the "leper's squint," the special window through which, without mingling with the congregation, they were permitted to witness the elevation of the Host, may still be seen as an interesting relic of the past. In all this, side by side with wise and necessary precaution, there was a certain element of inhumanity. But there would be no inhumanity in the practical isolation of a class of persons who retain, by their own choice, a liability to a disease far more loathsome and more contagious than leprosy, a liability from which they might at any time be released. We may live to see the formation of a Vaccination League, the members of which would refuse intercourse with the unvaccinated, would refuse to employ them in any capacity, to accept them as tenants, or pupils, or to reside in any quarters of towns in which they may be permitted to congregate. If the unvaccinated have "consciences," the vaccinated may be permitted to have them also, and scrupulously to follow their dictates. The form of conscience which leads its possessor to shun disease and those likely to be concerned in spreading it is undoubtedly more respectable than the form which claims the privilege of providing countless centres of infection.

